# Social Cognition and Socioecological Predictors of Home-Based Physical Activity Intentions, Planning, and Habits during the COVID-19 Pandemic

**DOI:** 10.3390/bs10090133

**Published:** 2020-08-31

**Authors:** Navin Kaushal, NiCole Keith, Susan Aguiñaga, Martin S. Hagger

**Affiliations:** 1 School of Health & Human Sciences, Department of Health Sciences, Indiana University, Indianapolis, IN 47405, USA; 2 School of Health & Human Sciences, Department of Kinesiology, Indiana University, Indianapolis, IN 47405, USA; nkeith@iupui.edu; 3 Department of Kinesiology and Community Health, University of Illinois at Urbana-Champaign, Urbana, IL 61820, USA; saguina2@illinois.edu; 4 Psychological Sciences, University of California, Merced, CA 95343, USA; mhagger@ucmerced.edu; 5 Faculty of Sport and Health Sciences, University of Jyväskylä, 40014 Jyväskylä, Finland

**Keywords:** physical activity, exercise equipment, home, habit, environment, pandemic, COVID-19

## Abstract

‘Shelter in place’ and ‘lockdown’ orders implemented to minimize the spread of COVID-19 have reduced opportunities to be physically active. For many, the home environment emerged as the only viable option to participate in physical activity. Previous research suggests that availability of exercise equipment functions as a determinant of home-based physical activity participation among the general adult population. The purpose of this study was to use a socioecological framework to investigate how the availability of exercise equipment at home predicts behavioral decisions, namely, intention, planning, and habits with respect to participation in physical activity. Participants (*n* = 429) were adults recruited in U.S. states subject to lockdown orders during the pandemic who completed measures online. A structural equation model indicated that availability of cardiovascular and strength training equipment predicted physical activity planning. Social cognition constructs mediated the relationship between each type of exercise equipment and intentions. Autonomous motivation and perceived behavioral control were found to mediate the relationship between each type of exercise equipment and habit. The availability of large cardiovascular and strength training equipment demonstrated significant predictive effects with intention, planning, habit, and autonomous motivation. Facilitating these constructs for home-based physical activity interventions could be efficacious for promoting physical activity.

## 1. Introduction

The health benefits of engaging in regular physical activity are well established [1]. Physical activity has been shown to enhance immune responses and serves as a buffer of psychosocial stressors [2]. Given the importance of immunity and effective stress management to health during the COVID-19 pandemic, maintaining physical activity levels may have utility in minimizing maladaptive physical and mental health conditions [3,4]. However, shelter-in-place and lockdown orders aimed at minimizing the spread of COVID-19 have disrupted the daily routine and habits of billions of people across the globe, including opportunities to be physically active. Many of these changes such as closures of gyms, recreation centers, and parks have created additional barriers for individuals to maintain a physically active lifestyle. Consequently, many people have been left with their homes as the only viable environment in which to engage in physical activity. Additionally, the shift of businesses to remote operations suggests that a large proportion of individuals will likely continue to work from home throughout the pandemic, and potentially post-pandemic. Though this shift is likely a favorable option for young families, as children will also be learning remotely from home. These circumstances demonstrate that the general adult population may spend a significant portion of their time at home. Identifying the determinants of home-based physical activity has, hence, become a public health priority and may provide evidence to inform interventions aimed at promoting home-based physical activity during the pandemic.

Investigation on how the home environment can be conducive to facilitating physical activity behavior is timely and relevant for the U.S. population during the pandemic. While investigation on the physical (micro) environment–behavior relationship has received considerable attention over the past decade for specific populations, such as adolescents [5] and older adults [6], the role of the home environment on physical activity in the general adult population still lags behind, with a dearth of theory-based research noted [7]. By contrast, there is considerable research applying social cognition theories, which focus on individual, belief-based constructs, in predicting health behavior [8], including physical activity [9]. The extant research on the environmental determinants of home-based physical activity has been largely informed by socioecological models, which identify proximal and distal socio-structural and physical environment factors that relate to physical activity [10,11]. In the current study we aimed to integrate variables of the physical environment derived from socioecological models alongside constructs from traditional social cognition theories to predict home-based physical activity.

### 1.1. An Integrated Model

A contemporary social cognition model of physical activity that could be extended to include environmental determinants is the integrated behavior change (IBC) model [12]. The IBC model was specifically designed to identify the predictors of physical activity behavior by integrating sets of core constructs from multiple social cognition and motivational models and relations among them [13]. The model proposes that physical activity participation is proximally predicted by motivational (intention), nonconscious constructs (habit), and the interaction between intention and planning. Intention is a function of attitudes, subjective norms, and perceived behavioral control as proposed by the theory of planned behavior [14]. Attitudes reflect an individuals’ personal belief that performing a particular behavior is worthwhile/beneficial. Subjective norms denote social beliefs that people who are influential would want the individual to perform the behavior. Finally, perceived behavioral control reflects beliefs that an individual has the control over performing the behavior, such as capacity, skill, and resources. The three determinants of intention are predicted by autonomous motivation, which reflects the extent to which the behavior is consistent with self-endorsed reasons for action and psychological needs [15].

Although there is considerable support for the effects of intention on health behaviors [16,17], a series of reviews have pointed out a substantive shortfall in individuals who do not successfully act on their intentions, thus suggesting post-intentional constructs that could influence this relationship [18,19,20,21,22]. A recognized post-intentional construct is action planning, which represents the extent to which an individual has created a plan that outlines when and where the behavior will be performed [23]. Action planning has also been shown to play a pivotal role in increasing the likelihood of successful intention enactment [24,25]. Based on this theory and research, the IBC model theorizes that forming an action plan facilitates the translation of intentions to behavior.

There is also recognition that initiation of some health behaviors, such as physical activity, are likely to be guided by automatic constructs, such as habit, in which behavior is enacted as a result of a predictable pattern [26,27]. Habits are developed through repeated experience of the behavior paired with the presentation of stable contexts or cues [27,28,29]. Findings support that we are able to differentiate between behaviors that are entirely consciously controlled (such as those with high level of complexity or performed at first time) and those that familiar and simple, which require lower cognitive resources [28,29]. The IBC model incorporates habit as a parallel determinant of behavior (intention), consistent with dual process theorizing [30]. Since behaviors guided by habit are predictable and hence performed with more ease [27], they are likely to be associated with perceptions that reflect familiarity and fluency, or a high perceived behavioral control. In addition, autonomous motivation reflects self-endorsed reasons for acting, and behaviors performed for autonomous reasons are likely to be performed regularly and support behavioral habits [29].

### 1.2. Environmental Determinants

A noted environmental determinant of home-based physical activity is the availability and use of exercise equipment. A linage of work derived from primary studies and systematic reviews has supported how the presence, size, and attributes of exercise equipment determine its regular use [7,31,32]. These findings suggest that individuals who have access to large exercise equipment at home (e.g., treadmill) were generally more likely to use them compared to smaller equipment (e.g., skipping rope), though correlations and predictive effect sizes were modest in magnitude. The type of large exercise equipment, which can be categorized as aerobic (cardiovascular training machines such as a treadmill or exercise bike) or anaerobic (strength training machines such as a portable gym or Bowflex), could yield further insight on how types of large exercise equipment contribute to physical activity participation. When analyzed using theoretical frameworks, research has found social cognitive constructs to mediate the relationship between socioecological variables and proximal behavioral determinants (e.g., intentions) [33,34]. In the present study we aimed to adopt a similar approach by incorporating availability of types of exercise equipment at home as an important physical environment determinant within the IBC model to predict home-based physical activity intentions and habits during the COVID-19 pandemic.

### 1.3. The Present Study

**Figure 1:**
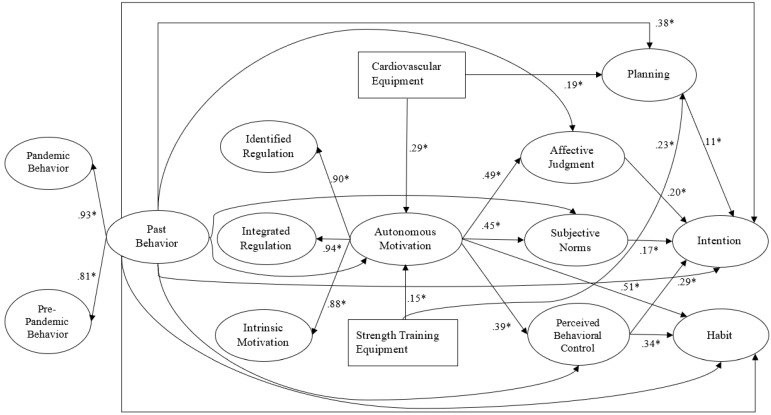
Note. Effect sizes are standardized parameter estimates from a structural equation model.* *p* < 0.001. Estimates for non-hypothesized pathways, including direct past behavior estimates, and demographic variables that controlled for model effects have been omitted for clarity.

The purpose of this study was to test the effects of two socioecological constructs, namely, availability and use of home exercise equipment (cardiovascular and strength training equipment), and constructs from the IBC model on intentions and habits with respect to home-based physical activity during the COVID-19 pandemic. Our proposed integrated model of home-based physical activity determinants is illustrated in Figure 1, and hypothesized effects within the model are summarized in Table 1. In line with the original model, we hypothesized that autonomous motivation would predict attitudes (H_1_), subjective norms (H_2_), perceived behavioral control (H_3_), and habit (H_4_) with respect to home-based physical activity. Attitudes (H_5_) and subjective norms (H_6_) were hypothesized to predict home-based physical activity intention, and perceived behavioral control would predict both intentions (H_7_) and habit (H_8_). In alignment with previous work [34,35], we expected social cognition and motivational constructs (autonomous motivation, attitudes, subjective norms, and perceived behavioral control) to mediate the relationship between past behavior and intentions (H_9_). We also expected determinants of habit (autonomous motivation and perceived behavioral control) to mediate the relationship between behavior and habit (H_10_). Consistent with previous research [36], we also proposed that planning would mediate the relationship between past behavior and intention (H_11_).

We also proposed hypotheses (H_12–19_) for the role of availability and use of home exercise equipment on home-based physical activity intentions and habits within the model. Consistent with recent findings [33,34], we predicted that the availability of cardiovascular (H_12_) and strength training (H_13_) equipment would facilitate autonomous motivation toward home-based physical activity. Availability of large exercise equipment was expected to be a fixed environmental determinant related to an individual’s home environment, which would be related to their exercise intentions and habit formation. Specifically, these variables were predicted to be an important source of information on which individuals based their judgements and motives for participating. For example, individuals’ beliefs toward home-based physical activity and autonomous motives to participate in home-based physical activity were expected to depend on the availability of relevant equipment, so we predicted these motives and beliefs would be related to the environmental constructs. In fact, such relations may reflect individuals’ motives to purchase or make the equipment available in the first place. As these motives and beliefs are integral to forming decisions to perform these behaviors in future, we hypothesized that autonomous motivation and the TPB constructs (attitudes, subjective norms, perceived behavioral control) would mediate the relationship between availability of cardiovascular and strength training equipment with intentions (H_14–15_). We also hypothesized that autonomous motivation, and the determinant of habit (perceived behavioral control), would mediate effects of availability of both types of exercise equipment on habit (H_16–17_), respectively. This hypothesis is consistent with research demonstrating that autonomous motivation and perceived behavioral control are associated with habit formation. While autonomous motivation is related to ongoing persistence with behaviors that leads to habit formation, a strong level of perceived behavioral control makes the behavior easier to perform, thus allowing habit to facilitate the behavior. The mediation effect reflects the role that exercise equipment availability drives self-determined motives and reduces the complexities to be active. Finally, availability and use of cardiovascular (H_18_) and strength training equipment (H_19_) were expected to be related to individuals’ plan to perform physical activity at home.

## 2. Materials and Methods

### 2.1. Participants and Recruitment

Participants (*n* = 429) were adults (age over 18), proficient in English, and residing in the United States with state-endorsed ‘lockdown’ measures in place for prevention of transmitting COVID-19 for May 2020. While definitions of lockdown/stay at home orders varied across the states, we specifically included states that had not reopened gyms and recreation centers/community centers at the time of recruitment. Recruitment was conducted using the Cloud Research Prime Panels service, which is a national recruitment platform [37]. Cloud Research is connected to more than 50 million users in the U.S. who indicated their interest to participate in survey research. Potential participants can also use a search feature on the website interface to view available studies that are recruiting participants. The studies are presented in tabs that provide the study title and identifying keywords. Individuals can then select the study link, which will take them to the first page of the survey and presents them with the study consent form. Individuals can then indicate their consent digitally and proceed to the survey if they decide to participate. Prime panel recruitment methods offered by Cloud Research have demonstrated strong scientific validity for recruiting samples with demographic profiles that correspond closely with those of the national population [38]. Additional details regarding Could Research’s Prime Panels can be found in Supplementary S1. Recruitment was only open to selected states that had gyms and recreation centers closed at the time, to control for these effects on home physical activity behavior. Participants were recruited in early May 2020 and resided in the following states: Connecticut, Michigan, Massachusetts, New Jersey, North Carolina, and Virginia, as well as Washington D.C. Participant data that showed noncompletion and survey scores that indicated inattentive responding (e.g., selecting same score on Likert-scale throughout the study) were discarded and replaced (*n* = 18). The study was approved by the university ethics committee (approval code #37764494) and informed consent was obtained from all participants.

### 2.2. Measures

The study adopted a cross-sectional design with participants completing commonly used, previously validated scaled measures adopted from previous research. All measures were framed in terms of expectations of engaging in physical activity over the next two weeks.

Theory of Planned Behavior Constructs. Participants completed standard measures of the social cognition constructs from the theory of planned behavior developed according to guidelines [14,39]. Specifically, participants completed multi-item measures of intention (e.g., “I intend to be regularly physically active”), attitudes (e.g., “For me, being physically active over the next two weeks could be wise”), subjective norms (e.g., “Most people who are important to me want me to engage in physical activity”), and perceived behavioral control (e.g., “I have complete control over whether I can engage in physical activity regularly”). Responses were provided on 5-point scales (e.g., 1 = strongly disagree and 5 = strongly agree). The TPB measures demonstrated good internal consistency: attitudes (α = 0.84), subjective norms (α = 0.95), perceived behavioral control (α = 0.82).

Planning. Planning was measured by encompassing both action and coping planning components [40,41]. The scale comprised three items (e.g., “I have made a detailed plan regarding when to exercise”) with responses provided on five-point scales (1 = strongly disagree and 5 = strongly agree). The scale demonstrated good internal consistency (α = 0.84).

Habit. Physical activity habit was measured using the self-report behavioral automaticity index (SRBAI; Gardner et al., 2012). A modified version of this scale was created to measure preparatory (exercise) habit, which demonstrated stronger predictive validity for exercise behavior [42]. Participants were provided with the following instructions “The preparatory exercise phase includes all the activities required to prepare you to engage in physical activity such as changing into your gym clothes. Please answer the following questions while keeping this definition in mind”. The instruction was followed by a common stem (“Preparing to participate in physical activity is something”) followed by the four SRBAI items (e.g., “I do without having to consciously remember”) with responses provided on five-point scales (1 = strongly disagree and 5 = strongly agree). The scale demonstrated strong internal consistency (α = 0.92).

Autonomous Motivation. Autonomous motivation was measured using the intrinsic (e.g., “I think it’s important to make the effort to engage in physical activity regularly”), identified (e.g., “I enjoy engaging in physical activity”), and integrated regulation (e.g., “I consider being physically active consistent with my values”; Ryan and Deci, 2017), subscales from the Behavioral Regulation and Exercise Questionnaire (BREQ-3) [43]. Responses were provided on five-point Likert scales (1 = strongly disagree and 5 = strongly agree). These subscales demonstrated strong reliability: intrinsic (α = 0.96), identified (α = 0.87), and integrated regulation (α = 0.93).

Exercise Equipment Availability. Participants were initially presented with the following two questions: “Do you own any large exercise cardio equipment such as a treadmill or a stationary bike?” and “Do you own any large strength training equipment such as a Bowflex machine, or a smith/squat machine?” with responses to each provided on a binary scale (1 = “yes” and 0 = “no”). Each question was followed by an open-ended question: “If you selected yes to the above question then please provide the type below” with responses provided in a free-response box. Participants’ responses to the open-ended question were coded to ensure participants had listed appropriate home gym equipment, providing a measure of fidelity of the exercise equipment availability measure.

Past Physical Activity Behavior. Past participation in physical activity was measured using the leisure-time exercise questionnaire. The measure comprises three open-ended items measuring time (duration and frequency) spent on type of exercise over the past two weeks at three intensities: mild, moderate, and strenuous. The measure has demonstrated good two-week test–retest reliability for the duration (r = 0.74) and frequency (r = 0.80) components [44]. This measure was administered twice. The first measure aimed to measure recent history of physical activity over the previous two weeks and was preceded by the following instruction: “We would like you to recall your average, weekly physical activity time over the past 2 weeks”. The second assessed participants physical activity levels prior to the pandemic and with the following instruction: “We would like you to recall your average, weekly, physical activity time prior to the COVID-19 Pandemic (when the economy was running as usual)”. For each assessment, we computed an aggregate score of physical activity in the moderate and vigorous intensities only.

### 2.3. Analysis Plan

Structural equation modeling [45] was used to estimate the fit and parameter estimates for the hypothesized effects in our proposed model (Figure 1). In the model, latent variables for each of the social cognition, motivation, and behavior constructs were indicated by their respective sets of items, and measures of availability of equipment and demographic variables were included as nonlatent variables. Autonomous motivation was created as a second order latent factor, comprising the three first-order latent variables for intrinsic, identified, and integrated motivation. Past behavior was a second order factor comprising first-order latent variables of pre-pandemic physical activity behavior and physical activity over the previous two weeks. The analysis was conducted using the SPSS AMOS 22.0 [46] with a full-information maximum likelihood estimation method. Analysis of missing data indicated that 98.7% cases were complete and missing data points were missing completely at random (MCAR χ^2^ = 80.31, df = 86, *p* = 0.65). Missing data were treated using hierarchical regression imputation before conducting the analyses [47]. Statistical power analyses for SEM were computed using Wolf et al.’s (2013) method, which considers the number of latent factors, factor indices, and factor loadings of the proposed model to estimate minimum sample size. The present model included six latent variables, with an average of three indicators per latent variable that yield an average factor loading of 0.86. The analysis returned a project minimum sample size of 225 participants would be required to test the model using a cross-sectional design. Model effects were controlled for demographic variables and past behavior, such that these variables predicted all constructs in the model. Overall fit of the proposed model was assessed by multiple criteria: the comparative fit index (CFI), Tucker–Lewis Index (TLI), the standardized root mean square residual (SRMR), and root mean square error of approximation (RMSEA). Values exceeding 0.90 and, preferably, approaching 0.95, for the CFI and TLI, and values less than 0.09 and 0.08 for the SRMSR and RMSEA, respectively, are indicative of acceptable fit of the model [48].

## 3. Results

### 3.1. Participant Characteristics

Participant characteristics of the sample are presented in Table 2. The mean age of participants was 47.1 years (*SD* = 16.26), 60% were female, and 53% of the sample identified themselves as being in either ‘very good’ or ‘excellent’ health. Participants reported participating in moderate-to-vigorous physical activity for an average of 152 min per week (*SD* = 148.3), within the past two weeks and reported 231 min (*SD* = 116.0) prior to the pandemic. Approximately 44% of the sample had a household income >$75,000 and 53% were married or in a common-law relationship.

### 3.2. Correlations and Preliminary Analyses

Zero-order latent variable correlations among the study variables are presented in Table 3. Large cardiovascular equipment significantly correlated with intention (*r* = 0.25, *p* < 0.001), planning (*r* = 0.31, *p* < 0.001), habit (*r* = 0.25, *p* < 0.001), and autonomous motivation (*r* = 0.27, *p* < 0.001). Strength training equipment statistically significantly correlated with intention (*r* = 0.16, *p* = 0.004), planning (*r* = 0.28, *p* < 0.001), habit (*r* = 0.27, *p* < 0.001), and autonomous motivation (*r* = 0.26, *p* < 0.001).

### 3.3. Structural Equation Model

Model fit indices indicated adequate fit of the proposed model (Figure 1) with the data according to the multiple criteria adopted, χ^2^ = 6762.55, df = 190, *p* < 001; CFI = 0.95; TLI = 0.94; RMSEA = 0.073, 95% CI RMSEA = 0.065, 0.080; SRMR = 0.066. Descriptive statistics and factor loadings for all model constructs are presented in Table 4. Standardized parameter estimates for the model are presented in Figure 1. With respect to hypothesis tests, autonomous motivation significantly predicted attitudes, (H_1_: β = 0.49, *p* < 0.001), subjective norms (H_2_: β = 0.45, *p* < 0.001), perceived behavioral control, (H_3_: β = 0.40, *p* < 0.001), and habit (H_4_: β = 0.51, *p* < 0.001). Direct predictors of intention included attitudes (H_5_: β = 0.20, *p* < 0.001), subjective norms (H_6_: β = 0.17, *p* < 0.001), and perceived behavioral control, (H_7_: β = 0.29, *p* < 0.001). Perceived behavioral control predicted habit (H_8_: β = 0.29, *p* < 0.001). Availability of cardiovascular (H_12_: β = 0.30, *p* < 0.001) and strength training equipment (H_13_: β = 0.17, *p* < 0.001) was also found to predict autonomous motivation. In addition, availability of cardiovascular (H_18_: β = 0.19, *p* < 0.001) and strength training (H_19_: β = 0.23, *p* < 0.001) equipment predicted planning for physical activity.

Social cognition constructs (attitudes, subjective norms, perceived behavioral control, and autonomous motivation) mediated the relationship between past behavior and intention (H_9_: β = 0.32, 95% CI 0.267, 0.374, *p* = 0.012). Autonomous motivation and perceived behavioral control also mediated the relationship between past behavior and habit (H_10_: β = 0.30, 95% CI 0.252, 0.339, *p* = 0.012). Future physical activity planning mediated the relationship between past behavior and intentions (H_11_: β = 0.08, 95% CI 0.045, 0.120, *p* = 0.005). Autonomous motivation and TPB determinants (attitudes, subjective norms, and perceived behavioral control) mediated the relationship between availability of cardiovascular equipment and intentions (H_14_: β = 0.20, 95% CI 0.140, 0.276, *p* = 0.015) and availability of strength training equipment and intentions (H_15_: β = 0.17, 95% CI 0.132, 0.211, *p* = 0.010). Autonomous motivation and perceived behavioral control were also found to mediate the relationship between cardiovascular equipment and habit (H_16_: β = 0.21, 95% CI 0.148, 0.257, *p* = 0.014) and strength training equipment and habit (H_17_: β = 0.13, 95% CI 0.090, 0.169, *p* = 0.009).

## 4. Discussion

The purpose of this study was to test an integrated model encompassing social cognition and motivational constructs related to the physical environment, specifically large home exercise equipment, as determinants of intentions and habits for participating in home-based physical activity during the COVID-19 pandemic. Hypothesized effects were formulated based on based on previous integrated models, such as the IBC model [12], and socio-ecological frameworks [10,11]. The study findings overall supported predictions of the IBC model in predicting home-based physical activity intentions, habits, and planning with the availability of exercise equipment. Social cognitive constructs (attitudes, subjective norms, perceived behavioral control, and autonomous motivation) mediated the relationship between exercise equipment and physical activity intentions. Similarly, habit determinants (perceived behavioral control and autonomous motivation) mediated the relationship between exercise equipment and physical activity habit. Finally, exercise equipment availability and use directly predicted physical activity planning. Overall, availability of home exercise equipment demonstrated predictive effects on proximal determinants of physical activity (intentions and habits) and post-intentional processes (physical activity planning).

The current model provided evidence for the social cognition and motivational determinants of intentions and habits to participate in home-based physical activity that aligns with previous theory-based research. Specifically, we found indirect effects of autonomous motivation on intentions to participate in home-based physical activity was mediated by the social cognition constructs from the TPB: attitudes, subjective norms, and perceived behavioral control. These findings are consistent with the IBC model and a considerable body of previous research integrating constructs from SDT and TPB [49]. The effect sizes of intention determinants are in the range with meta-analytic studies that highlight the importance of these belief-based constructs in predicting physical activity intentions [50]. Importantly, habit was predicted by autonomous motivation, which corroborates with previous work, suggesting that autonomously motivated individuals are more likely to report experiencing their behavior as habitual [51]. This is consistent with theory suggesting that autonomous motivation tends to motivate repeated performance of a behavior, often in stable unchanging contexts, which are ideal circumstances for habit development. Consistent with SDT, the underlying mechanism behind their persistence may be because autonomously motivated individuals perceive the behavior as an opportunity to satisfy their psychological needs, and, therefore, are more likely to persistently seek need-satisfying behaviors. Habit was also found to be predicted by perceived behavioral control, which also builds on previous research and theory that suggests the development of habits tends to occur in those who view themselves as having high capacity to perform the behavior and perceive fewer barriers [27,52,53]. Finally, past behavior was found to simultaneously predict intentions and habit via the social cognition constructs, which is in accordance with the predictions of dual process theories [26,30], that is past behavior is a proxy for habits as it establishes familiarity, and past experience is used to estimate intention to perform future behavior [17,54]. Taken together, the current findings with respect to the psychological determinants of intentions to participate in home-based physical activity are consistent with predictions of the IBC model, and with its component theories, and suggests that this is a viable framework to understand the determinants of intentions to perform home-based physical activity.

A highly unique aspect of the current research was the inclusion of constructs representing the physical environment, namely availability and use of cardiovascular and strength training equipment as additional determinants in the IBC model. We predicted that equipment availability in the home would be implicated in the processes that predict intentions and habits in the current model. Specifically, we expected effects of exercise equipment on intentions and habits to be mediated by the social cognition and motivational determinants of behavior. Supporting our predictions, autonomous motivation and TPB constructs mediated the relationship between the availability of exercise equipment and intentions and habits. These findings support recent work on how motivational constructs account for effects of socio-structural constructs on intentions and habit [33,34]. The findings also support these effects in the context of home-based physical activity, and focus on variables that reflect elements of the environment likely to be relevant to decision making and habit formation [7,31,32]. The mediation effects suggest that the availability and use of exercise equipment are important inputs for informing decisions to exercise at home, and are also important for habit formation [50].

We also hypothesized that availability of exercise equipment would facilitate the development of planning, and the results supported our predictions. The importance of planning to perform a behavior regularly are well documented [55,56,57], however, little work has investigated necessary resources that facilitate effective planning strategies, specifically in the home environment [58,59,60]. The present study findings are pertinent since planning demonstrated a critical role in mediating the intention–behavior pathway, which aligns with previous research [36]. Consequently, these findings may also highlight socioeconomic inequity as those with lower financial resources could be at a disadvantage at owning large exercise equipment, though dedicated hypotheses and tests are warranted for investigating those barriers. While the literature on the home environment support effects of larger exercise equipment in motivating plans to perform home physical activity participation [7,31,32], future research should investigate whether smaller, or location-movable workout equipment (e.g., pullup bars, skipping rope) are effective in promoting plans to be active at home.

As studies continue to emerge indicating the importance of remaining physically active during the pandemic [61,62,63], the current study offered a unique perspective by investigating the role of home exercise equipment. Though the study findings should be kept in an appropriate scope along with its limitations and notes for improvement. The cross-sectional analysis limits us from understanding change of determinants across time, and hence the study can be improved by employing a longitudinal design. Additionally, while device-assessed physical activity (e.g., wearables/accelerometers) would strengthen the findings, the university ethics committee at the time of data collection did not permit in-person/use of wearable devices in order to prevent transmission of the virus. Only measures that could be administered remotely and online were permitted during the pandemic. Finally, the current study could be improved by assessing the presence of children and dependents at home as these socio-structural variables could function as potential moderators between exercise equipment and their determinants. Future work that conducts condition/group analyses to compare behavioral determinants between individuals owning either type and both types of equipment might highlight specific scenarios where some behavioral determinants could be more detrimental than others.

Despite these limitations, the study provides notable strengths and novel contributions. The home presents a potentially conducive environment for physical activity for those under ‘lockdown’, as it also represents an environment that we are able to modify and control. The timing of this study offered a unique insight into the social cognitive, motivational, and environmental constructs related to physical activity behavior at home when access to gyms and recreation facilities was completely barred. It is also worth noting that physical activity guidelines promoted by American College of Sports Medicine include both aerobic and strength training exercises for the general population [64]. However, there is a disproportionate amount of research that focuses on promotion of cardiovascular exercise compared to resistance training in the literature [65]. The current investigation and findings pertaining to the promotion of strength training exercises contributes to an area in the literature that warrants greater attention.

## 5. Conclusions

As more businesses shift to remote work practices post-pandemic, a greater proportion of individuals will likely work from home, thus emphasizing the importance of understanding how determinants in the home environment predict physical activity levels. The present study found supported proposed relations between variables from social cognition, motivational, and socio-ecological theories that add insight to home-based physical activity intentions and habits. In summary, both, cardiovascular and strength training equipment demonstrated similar predictive effects within the model. Equipment effects on proximal intentional (perceived behavioral control and affective judgement), in addition to behavioral determinants (planning and habit), providing valuable notes for designing randomized controlled trials. Indirect effects from exercise equipment to perceived behavioral control suggests that interventions should ensure that their participants have the skill and ability to use exercise equipment they may receive. Similarly, indirect effects from exercise equipment to affective judgement suggest that the provided equipment should be enjoyable for the participants to use. Additionally, assisting participants to develop exercise plans specific to the type of equipment could help translate their exercise intentions to behavior. Finally, exercise equipment was found to have an indirect effect on habit, which suggests that helping participants implement habit formation techniques could make their exercise behavior easier to initiate.

## Figures and Tables

**Table 1:** Summary of study hypotheses.

Hypothesis	Independent Variable	Dependent Variable	Proposed Mediator(s)
H_1_	Autonomous Motivation	Attitudes	
H_2_	Autonomous Motivation	Subjective Norms	
H_3_	Autonomous Motivation	Perceived Behavioral Control	
H_4_	Autonomous Motivation	Habit	
H_5_	Attitudes	Intention	
H_6_	Subjective Norms	Intention	
H_7_	Perceived Behavioral Control	Intention	
H_8_	Perceived Behavioral Control	Habit	
H_9_	Past Behavior	Intention	Autonomous Motivation, Attitudes, Subjective Norms, Perceived Behavioral Control
H_10_	Past Behavior	Habit	Autonomous Motivation, Perceived Behavioral Control
H_11_	Past Behavior	Intention	Planning
H_12_	Cardiovascular Equipment	Autonomous Motivation	
H_13_	Strength Training Equipment	Autonomous Motivation	
H_14_	Presence of Cardiovascular Equipment	Intention	Autonomous Motivation, Attitudes, Subjective Norms, Perceived Behavioral Control
H_15_	Presence of Strength Training Equipment	Intention	Autonomous Motivation, Attitudes, Subjective Norms, Perceived Behavioral Control
H_16_	Cardiovascular Equipment	Habit	Autonomous Motivation, Perceived Behavioral Control
H_17_	Strength Training Equipment	Habit	Autonomous Motivation, Perceived Behavioral Control
H_18_	Cardiovascular Equipment	Planning	
H_19_	Strength Training Equipment	Planning	

**Table 2:** Demographic characteristics of the sample.

Characteristic	Value
Age, *M* (*SD*)	*M* = 47.05 (*SD* = 16.26)
Female, %	60.0
Household Income, %	
<$50,000	37.7
$50,001–$75,000	17.1
$75,001–$100,001	15.7
$100,001–$150,000	15.0
$150,000–$200,000	7.2
>$200,000	6.0
Job Status, %	
Homemaker	7.2
Retired	19.4
Student	7.2
Social welfare	0.7
Temporarily unemployed	11.5
Full-time employed	42.5
Part-time employment	11.3
Education, %	
Less than high school	1.6
Highschool	4.6
College diploma	15
Some University	17.8
Bachelor’s degree	32.1
Master’s degree	17.6
Earned doctorate	3.8
Graduate/Professional Degree	1.6
Marital Status, %	
Never married	32.8
Married/common law	52.9
Separated/divorced/widowed	13.4
Overall Self-rated Health, %	
Excellent	17.6
Very Good	35.1
Good	33
Fair	12.7
Poor	1.6

**Table 3:** Zero-order correlations among study variables.

Construct	1	2	3	4	5	6	7	8	9	10
1. Intention	–	0.37 ***	0.32 ***	0.46 ***	0.45 ***	0.54 ***	0.54 ***	0.25 ***	0.16 **	0.49 ***
2. Planning		–	0.58 ***	0.18 ***	0.37 ***	0.33 ***	0.63 ***	0.31 ***	0.28 ***	0.40 ***
3. Habit			–	0.14 **	0.20 ***	0.27 ***	0.60 ***	0.25 ***	0.27 ***	0.34 ***
4. Attitudes				–	0.52 ***	0.56 ***	0.46 ***	0.06	0.09	0.25 ***
5. Subjective Norms					–	0.52 ***	0.49 ***	0.05	0.03	0.27 ***
6. Perceived Behavioral Control						–	0.61 ***	0.11 *	0.07	0.42 ***
7. Autonomous Motivation							–	0.27 ***	0.13 **	0.53 ***
8. Cardio equipment								–	0.26 ***	0.19 **
9. Strength training equipment									–	0.01
10. Behavior										–

Note. The table presents zero-order bivariate and model correlations (values within brackets). Behavior = physical activity performed at moderate-to-vigorous intensity. * *p* < 0.05 ** *p* < 0.01 *** *p* < 0.001.

**Table 4:** Means, standard deviations, and factor loadings of study items.

Indicator	Factor Loadings	Means/SD
1. Perceived Behavior Control.1	0.72	4.08 (0.89)
2. Perceived Behavior Control.2	0.73	4.04 (0.92)
3. Perceived Behavior Control.3	0.75	3.71 (1.09)
4. Attitudes.1	0.93	4.08 (0.83)
5. Attitudes.2	0.84	3.00 (1.09)
6. Attitudes.3	0.92	4.00 (0.82)
7. Subjective Norms.1	0.91	3.57 (0.61)
8. Subjective Norms.2	0.95	3.84 (0.97)
9. Subjective Norms.3	0.91	3.54 (1.02)
10. Habit.1	0.82	3.33 (3.36)
11. Habit.2	0.93	3.22 (3.03)
12. Habit.3	0.89	3.14 (1.18)
13. Habit.4	0.74	2.92 (1.73)
14. Autonomous Motivation-Intrinsic	0.90	3.85 (0.85)
15. Autonomous Motivation- Integrated	0.94	3.33 (1.04)
16. Autonomous Motivation- Identified	0.88	3.29 (1.08)
17. Behavior Pandemic	0.93	152.20 (148.25)
18. Behavior Pre-Pandemic	0.81	231.86 (116.02)

Note. Behavior = moderate-to-vigorous intensity physical activity.

## Supplementary Materials

The following are available online at https://www.mdpi.com/2076-328X/10/9/133/s1, Cloud Research. Reference [38] is cited in the supplementary.

## References

[1] Riebe D., Ehrman J.K., Liguori G., Magal M. (2018). American College of Sports Medicine: Guidelines for Exercise Testing and Prescription.

[2] Penedo F.J., Dahn J.R. (2005). Exercise and well-being: A review of mental and physical health benefits associated with physical activity. Curr. Opin. Psychiatry.

[3] Chen P., Mao L., Nassis G.P., Harmer P., Ainsworth B.E., Li F. (2020). Coronavirus disease (COVID-19): The need to maintain regular physical activity while taking precautions. J. Sport Health Sci..

[4] Holmes E.A., O’Connor R.C., Perry V.H., Tracey I., Wessely S., Arseneault L., Ballard C., Christensen H., Cohen Silver R., Everall I. (2020). Multidisciplinary research priorities for the COVID-19 pandemic: A call for action for mental health science. Lancet Psychiatry.

[5] Gao Z., Chen S., Pasco D., Pope Z. (2015). A meta-analysis of active video games on health outcomes among children and adolescents. Obes. Rev..

[6] Larsen L.H., Schou L., Lund H.H., Langberg H. (2013). The physical effect of exergames in healthy elderly—A systematic review. Games Health J..

[7] Kaushal N., Rhodes R.E. (2014). The home physical environment and its relationship with physical activity and sedentary behavior: A systematic review. Prev. Med..

[8] Conner M., Norman P. (2015). Predicting and Changing Health Behaviour: Research and Practice with Social Cognition Models.

[9] Rhodes R.E., McEwan D., Rebar A.L. (2018). Theories of physical activity behaviour change: A history and synthesis of approaches. Psychol. Sport Exerc..

[10] Sallis J.F., Owen N., Fisher E. (2015). Ecological Models of Health Behavior.

[11] Salmon J., Hesketh K.D., Arundell L., Downing K.L., Biddle S.J.H., Hagger M.S., Cameron L.D., Hamilton K., Hankonen N., Lintunen T. (2020). Changing behavior using ecological models. The Handbook of Behavior Change.

[12] Hagger M.S., Chatzisarantis N.L.D. (2014). An integrated behavior change model for physical activity. Exerc. Sport Sci. Rev..

[13] Hagger M.S., Chatzisarantis N.L.D. (2009). Integrating the theory of planned behaviour and self-determination theory in health behaviour: A meta-analysis. Br. J. Health Psychol..

[14] Ajzen I. (1991). The theory of planned behavior. Organ. Behav. Hum. Decis. Process..

[15] Deci E., Ryan R. (2002). Handbook of Self-Determination Research.

[16] McEachan R.R.C., Conner M., Taylor N.J., Lawton R.J. (2011). Prospective prediction of health-related behaviours with the Theory of Planned Behaviour: A meta-analysis. Health Psychol. Rev..

[17] Hagger M.S., Polet J., Lintunen T. (2018). The reasoned action approach applied to health behavior: Role of past behavior and tests of some key moderators using meta-analytic structural equation modeling. Soc. Sci. Med..

[18] Sheeran P., Webb T.L. (2016). The intention–behavior gap. Soc. Personal. Psychol. Compass.

[19] Hagger M.S. (2010). Health psychology review: Advancing theory and research in health psychology and behavioural medicine. Health Psychol. Rev..

[20] Orbell S., Sheeran P. (1998). ‘Inclined abstainers’: A problem for predicting health-related behaviour. Br. J. Soc. Psychol..

[21] Rhodes R.E. (2014). Bridging the physical activity intention-behaviour gap: Contemporary strategies for the clinician. Appl. Physiol. Nutr. Metab..

[22] Rhodes R.E., De Bruijn G.J. (2013). How big is the physical activity intention-behaviour gap? A meta-analysis using the action control framework. Br. J. Health Psychol..

[23] Gollwitzer P.M. (1999). Implementation intentions: Strong effects of simple plans. Am. Psychol..

[24] Hagger M.S., Luszcynska A. (2014). Implementation intention and action planning interventions in health contexts: State of the research and proposals for the way forward. App. Psychol. Health Well-Being.

[25] Bélanger-Gravel A., Godin G., Amireault S. (2013). A meta-analytic review of the effect of implementation intentions on physical activity. Health Psychol. Rev..

[26] Ouellette J.A., Wood W. (1998). Habit and intention in everyday life: The multiple processes by which past behavior predicts future behavior. Psychol. Bull..

[27] Wood W., Rünger D. (2015). Psychology of habit. Annu. Rev. Psychol..

[28] Gardner B. (2015). A review and analysis of the use of ‘habit’ in understanding, predicting and influencing health-related behaviour. Health Psychol. Rev..

[29] Hagger M.S. (2019). Habit and physical activity: Theoretical advances, practical implications, and agenda for future research. Psychol. Sport Exerc..

[30] Evans J.S.B.T. (2008). Dual-processing accounts of reasoning, judgment, and social cognition. Annu. Rev. Psychol..

[31] Swartz M.C., Lewis Z.H., Lyons E.J., Jennings K., Middleton A., Deer R.R., Arnold D., Dresser K., Ottenbacher K.J., Goodwin J.S. (2017). Effect of home- and community-based physical activity interventions on physical function among cancer survivors: A systematic review and meta-analysis. Arch. Phys. Med. Rehabil..

[32] Claes J., Buys R., Budts W., Smart N., Cornelissen V.A. (2017). Longer-term effects of home-based exercise interventions on exercise capacity and physical activity in coronary artery disease patients: A systematic review and meta-analysis. Eur. J. Prev. Cardiol..

[33] Hagger M.S., Hamilton K. (2020). Effects of socio-structural variables in the theory of planned behavior: A mediation model in multiple samples and behaviors. Psychol. Health.

[34] Olson J.L., Ireland M.J., March S., Biddle S.J.H., Hagger M.S. (2019). Physical activity in peri-urban communities: Testing intentional and implicit processes within an ecological framework. Appl. Psychol. Health Well-Being.

[35] Brown D.J., Hagger M.S., Hamilton K. (2020). The mediating role of constructs representing reasoned-action and automatic processes on the past behavior-future behavior relationship. Soc. Sci. Med..

[36] Girelli L., Hagger M., Mallia L., Lucidi F. (2016). From perceived autonomy support to intentional behaviour: Testing an integrated model in three healthy-eating behaviours. Appetite.

[37] Cloud Research Homepage. https://www.cloudresearch.com/.

[38] Chandler J., Rosenzweig C., Moss A.J., Robinson J., Litman L. (2019). Online panels in social science research: Expanding sampling methods beyond Mechanical Turk. Behav. Res. Methods.

[39] Ajzen I. (2002). Perceived behavioral control, self-efficacy, locus of control, and the theory of planned behavior. J. Appl. Soc. Psychol..

[40] Sniehotta F.F., Scholz U., Schwarzer R. (2005). Bridging the intention-behaviour gap: Planning, self-efficacy, and action control in the adoption and maintenance of physical exercise. Psychol. Health.

[41] Sniehotta F.F., Scholz U., Schwarzer R. (2006). Action plans and coping plans for physical exercise: A longitudinal intervention study in cardiac rehabilitation. Br. J. Health Psychol..

[42] Kaushal N., Rhodes R.E., Meldrum J.T., Spence J.C. (2017). The role of habit in different phases of exercise. Br. J. Health Psychol..

[43] Markland D., Tobin V. (2004). A modification to the behavioural regulation in exercise questionnaire to include an assessment of amotivation. J. Sport Exerc. Psychol..

[44] Godin G., Shephard R.J., Colantonio A. (1986). The cognitive profile of those who intend to exercise but do not. Public Health Rep..

[45] Enders C.K. (2011). Analyzing longitudinal data with missing values. Rehabil. Psychol..

[46] Arbuckle J.L. (2014). Amos (Version 23.0) [Computer Program].

[47] Awang Z. (2012). Structural Equation Modeling Using Amos Graphic.

[48] Marsh H.W., Hau K.-T., Wen Z. (2004). In search of golden rules: Comment on hypothesis-testing approaches to setting cutoff values for fit indexes and dangers in overgeneralizing Hu and Bentler’s (1999) findings. Struct. Equ. Model. A Multidiscip. J..

[49] Chan D.K.C., Zhang L., Lee A.S.Y., Hagger M.S. (2020). Reciprocal relations between autonomous motivation from self-determination theory and social cognition constructs from the theory of planned behavior: A cross-lagged panel design in sport injury prevention. Psychol. Sport Exerc..

[50] Hagger M.S., Chan D.K.C., Protogerou C., Chatzisarantis N.L.D. (2016). Using meta-analytic path analysis to test theoretical predictions in health behavior: An illustration based on meta-analyses of the theory of planned behavior. Prev. Med..

[51] Radel R., Pelletier L., Pjevac D., Cheval B. (2017). The links between self-determined motivations and behavioral automaticity in a variety of real-life behaviors. Motiv. Emot..

[52] Kaushal N., Rhodes R.E. (2015). Exercise habit formation in new gym members: A Longitudinal study. J. Behav. Med..

[53] Verplanken B., Melkevik O. (2008). Predicting habit: The case of physical exercise. Psychol. Sport Exerc..

[54] Chatzisarantis N.L., Hagger M.S., Smith B., Phoenix C. (2004). The influences of continuation intentions on execution of social behaviour within the theory of planned behaviour. Br. J. Soc. Psychol..

[55] Koh L.H., Hagger M.S., Goh V.H.H., Hart W.G., Gucciardi D.F. (2017). Effects of a brief action and coping planning intervention on completion of preventive exercises prescribed by a physiotherapist among people with knee pain. J. Sci. Med. Sport.

[56] Gollwitzer P.M., Sheeran P. (2006). Implementation intentions and goal achievement: A meta-analysis of effects and processes. Advances in Experimental Social Psychology.

[57] Dombrowski S.U., Endevelt R., Steinberg D.M., Benyamini Y. (2016). Do more specific plans help you lose weight? Examining the relationship between plan specificity, weight loss goals, and plan content in the context of a weight management programme. Br. J. Health Psychol..

[58] Ashworth N.L., Chad K.E., Harrison E.L., Reeder B.A., Marshall S.C. (2005). Home versus center based physical activity programs in older adults. Cochrane Database Syst. Rev..

[59] Aoike D.T., Baria F., Kamimura M.A., Ammirati A., de Mello M.T., Cuppari L. (2015). Impact of home-based aerobic exercise on the physical capacity of overweight patients with chronic kidney disease. Int. Urol. Nephrol..

[60] Dondzila C.J., Swartz A.M., Keenan K.G., Harley A.E., Azen R., Strath S.J. (2016). Translating exercise interventions to an in-home setting for seniors: Preliminary impact on physical activity and function. Aging Clin. Exp. Res..

[61] Zbinden-Foncea H., Francaux M., Deldicque L., Hawley J.A. (2020). Does high cardiorespiratory fitness confer some protection against pro-inflammatory responses after infection by SARS-CoV-2?. Obesity.

[62] Rahmati-Ahmadabad S., Hosseini F. (2020). Exercise against SARS-CoV-2 (COVID-19): Does workout intensity matter? (A mini review of some indirect evidence related to obesity). Obes. Med..

[63] Diamond R., Waite F. (2020). Physical activity in a pandemic: A new treatment target for psychological therapy. Psychol. Psychother. Theory Res. Pract..

[64] (2017). American College of Sports Medicine Guidelines for Exercise Testing and Prescription.

[65] Rhodes R.E., Lubans D.R., Karunamuni N., Kennedy S., Plotnikoff R. (2017). Factors associated with participation in resistance training: A systematic review. Br. J. Sports Med..

