# Supplementary material for: Social Cognition and Socioecological Predictors of Home-Based Physical Activity Intentions, Planning, and Habits during the COVID-19 Pandemic
Source: Behav Sci (Basel). 2020 Aug 31;10(9):133. doi: 10.3390/bs10090133 (PMC7551445; doi:10.3390/bs10090133)
Supplement: Supplementary file 1 [file behavsci-10-00133-s001.pdf]

Supplementary File S1

Cloud Research is a data provision /marketing company that hosts online panels of members of the general public for marketing and research. Prime Panels is a system used by the company that aggregates dozens of online research panels to provide greater sampling power compared with single panel recruitment platforms. Each panel within the system has its own participant pool. Participants from these panels are profiled on hundreds of demographic variables and variables representing their interests and preferences. This information is used to send invitations to potential participants based on the study's eligibility criteria. Prime Panels has access to more than 50 million participants across the United States which allows the system to recruit samples representing the general national population to highly specific population groups [38].
